# Translation, Content Validity, and Psychometric Evaluation of the Personality Inventory for DSM-5 Brief Form (PID-5-BF) in Standard Arabic

**DOI:** 10.3390/healthcare14131917

**Published:** 2026-07-01

**Authors:** Saleh A. Alghamdi, Anas Alrasheed, Abdulrahman Kariri, Osama Alghamdi, Muhammad Shakir Raza

**Affiliations:** 1 Department of Psychiatry, College of Medicine, Imam Mohammad Ibn Saud Islamic University (IMSIU), Riyadh 11564, Saudi Arabia; 2 College of Medicine, Imam Mohammad Ibn Saud Islamic University (IMSIU), Riyadh 11564, Saudi Arabia; anas.m.alrsheed@gmail.com (A.A.);; 3 Doctor Raza Medical Research Consultancy, Sargodha 40100, Pakistan; doctorshakirraza@gmail.com

**Keywords:** personality disorder, PID-5-BF, Arabic, psychometrics, confirmatory factor analysis, DSM-5 AMPD, content validity, Saudi Arabia

## Abstract

**Background:** Arabic-speaking populations lack a brief, psychometrically evaluated instrument for assessing maladaptive personality traits within the DSM-5 Alternative Model for Personality Disorders (AMPD). Objective: To translate the Personality Inventory for DSM-5—Brief Form (PID-5-BF) into Standard Arabic and evaluate its content validity, reliability, and factor structure in a Saudi community sample. **Methods:** The PID-5-BF was translated through a multistep forward–backward procedure with expert panel review. Twenty-five pilot participants rated item clarity and simplicity. The final Arabic version was administered to 328 Saudi adults, and 52 verified pairs completed it twice over a mean interval of 16.1 days. Confirmatory factor analysis (CFA) tested the a priori five-factor model. Principal axis factoring with Promax rotation and Horn’s parallel analysis were used to examine factor retention. Reliability indices included Cronbach’s alpha and intraclass correlation coefficients [ICC(2,1), absolute agreement]. **Results:** Content validity was excellent (S-CVI/Ave = 0.93 for clarity; 0.94 for simplicity). CFA showed an acceptable RMSEA (0.068, 90% CI 0.062–0.075) but incremental fit below thresholds (CFI = 0.84; TLI = 0.82; SRMR = 0.12). Parallel analysis retained four factors. Domain alphas ranged from 0.70 to 0.78 (total = 0.89), and domain ICCs were 0.74 to 0.85 (total = 0.88). Antagonism items showed 50–58% floor effects. **Conclusions:** The standard-Arabic PID-5-BF shows acceptable content validity, internal consistency, and temporal stability, with partial structural support. Further work using ordinal estimation, measurement invariance testing, and external validity assessment is needed before routine clinical adoption.

## 1. Introduction

A personality disorder (PD) is a persistent and inflexible pattern of inner experience and behaviour that deviates markedly from the norms and expectations of an individual’s culture, is pervasive, has its onset in adolescence or early adulthood, is stable over time, and results in distress or impairment [1]. In a meta-analysis, Winsper et al. estimated the worldwide pooled prevalence of any PD to be 7.8%, with 3.8% for Cluster A (odd/eccentric) disorders, 2.8% for Cluster B disorders (dramatic/erratic), and 5.0% for Cluster C disorders (anxious/fearful) [2]. The association between PDs and other mental illnesses is marked by substantial comorbidity, shared risk factors, and reciprocal interactions, with PDs increasing the severity and chronicity of multiple psychiatric conditions [3,4]. PDs have also been linked to poorer quality of life and greater medical comorbidity, demonstrating their clinical and public health relevance [5].

While personality disorders have traditionally been conceptualised using the categorical diagnoses in DSM-5 Section II, the DSM-5 also includes an Alternative Model for Personality Disorders (AMPD) in Section III that adopts a dimensional framework [1]. Within the AMPD, personality pathology is described in terms of impairments in self and interpersonal functioning (Criterion A) and maladaptive personality traits (Criterion B), the latter operationalised through five broad domains—negative affectivity, detachment, antagonism, disinhibition, and psychoticism—and their constituent facets [1,6]. The Personality Inventory for DSM-5 (PID-5), a 220-item self-report measure, was developed to assess these Criterion B trait domains and has been translated into multiple languages [6,7]. To reduce respondent burden, the PID-5—Brief Form (PID-5-BF) was developed as a 25-item condensed version that assesses the same five higher-order domains, with five items each [8,9].

Personality disorders remain under-recognised in many Arabic-speaking settings, where brief, standardised, culturally appropriate assessment tools are limited. An Arabic version of the full 220-item PID-5 has been validated in a community sample from the United Arab Emirates [10]; however, that work used the long form and a specific regional variety of Arabic. In Saudi settings, there remains a practical need for a brief instrument written in Standard Arabic that is suitable for routine clinical use and large surveys across regions and educational backgrounds. A standard-Arabic PID-5-BF could improve comparability with international studies and support the integration of dimensional trait assessment into research and practice. The same dataset analysed here was previously used to examine the sociodemographic correlates of maladaptive personality traits in Saudi adults [11]; however, the present study addresses a distinct objective focused on the translation, content validity, reliability, and structural evaluation of the Arabic PID-5-BF.

## 2. Study Aims and Hypotheses

This study aimed to translate, culturally adapt, and psychometrically evaluate the PID-5-BF in Standard Arabic in a Saudi community sample. Because the original instrument has an established five-domain theoretical structure, we tested the a priori five-factor model using confirmatory factor analysis (CFA) and used exploratory factor analysis (EFA) and Horn’s parallel analysis as complementary structural checks. We hypothesised that the Arabic PID-5-BF would (1) demonstrate adequate content validity as judged by Arabic-speaking adults; (2) replicate the a priori five-factor structure; (3) show acceptable internal consistency (α ≥ 0.70) for each domain; and (4) demonstrate acceptable short-term test–retest reliability in a subsample of participants.

## 3. Materials and Methods

### 3.1. Study Design and Source Data

This was an online psychometric study with three linked components: cultural adaptation and pilot testing, a short-term test–retest substudy, and a main community administration. Data collection proceeded in four sequential phases through a single online form: pilot cognitive debriefing (*n* = 25), first test–retest administration (*n* = 63), second test–retest administration approximately two weeks later (*n* = 58), and main community administration (*n* = 285).

### 3.2. Participants and Eligibility

Eligibility for the main analytic sample required informed consent, Saudi nationality, an age of 18 years or older, and complete age data. Participants were required to have completed all 25 PID-5-BF items and the core sociodemographic questions (age, gender, marital status, education level, employment/field of study, and region of residence). Recruitment was conducted online via social media and messaging applications; participation was voluntary and uncompensated.

### 3.3. Data Quality Criteria

**Figure 1:**
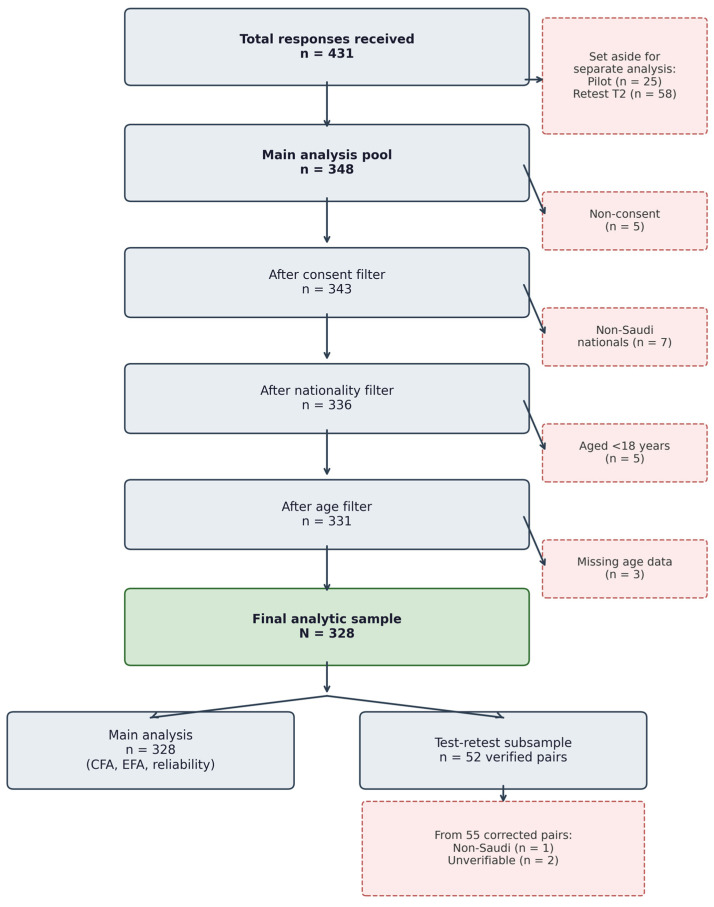
Participant flow diagram.

Data quality exclusions were kept separate from the eligibility criteria. Records were excluded during cleaning if consent was not provided (*n* = 5), nationality was not Saudi (*n* = 7), age was below 18 years (*n* = 5), or age data were missing (*n* = 3). The pilot cognitive debriefing sample (*n* = 25) and the second retest administration (*n* = 58) were analysed separately. Item responses recorded as Arabic text labels were recoded to the standard 0–3 numeric scale using a documented mapping verified against the original PID-5-BF response anchors [8]. The participant flow is presented in Figure 1.

### 3.4. Sample Size Considerations

The analytic sample of N = 328 participants exceeded psychometric guidelines recommending approximately 10 participants per item and a minimum of 200–300 cases for factor analysis [12]. With 25 items, this sample size was adequate for CFA, EFA, reliability estimation, and parallel analysis. The analytic sample in the present study (N = 328) differs from the N = 343 reported in an earlier paper using the same dataset [11]. The present analysis applied stricter exclusion criteria, namely the separation of the pilot cognitive debriefing sample (*n* = 25) and the second test–retest administration (*n* = 58) from the main community data, together with the consistent application of the eligibility criteria specified in Section 3.2 and the data quality criteria specified in Section 3.3.

### 3.5. Instrument and Scoring

The PID-5-BF is a 25-item self-report measure of maladaptive personality traits rated on a four-point scale from 0 (very false or often false) to 3 (very true or often true) [8]. The instrument yields five domain scores based on five items each, with domain scores ranging from 0 to 15. In this study, domain scores were computed using the original APA item-to-domain mapping, in which domain assignments are non-sequential across the 25 items [8]. A total score (range: 0–75) was computed by summing all items and treated only as a secondary descriptive index of shared maladaptive trait burden, not as evidence of unidimensionality.

### 3.6. Translation and Cultural Adaptation

The translation followed a multistep forward–backward approach consistent with published cross-cultural adaptation guidelines [13,14]. The PID-5-BF is copyrighted by the American Psychiatric Association (APA), which permits reproduction for clinical and research use without prior written permission [8]. The process comprised five stages: (1) two independent bilingual clinicians with experience in psychometric assessment in Saudi Arabia produced forward translations into Standard Arabic; (2) an expert panel of bilingual clinicians familiar with DSM-5 terminology reconciled both versions into a consensus draft, selecting Standard Arabic wording understood across Saudi regions and avoiding dialect-specific expressions; (3) two translators blinded to the original independently back-translated the consensus Arabic version; (4) two bilingual experts compared both back-translations with the original and resolved discrepancies by consensus; and (5) the pre-final Arabic version was pilot-tested with 25 Arabic-speaking adults, who completed the scale and rated each item for clarity and simplicity.

### 3.7. Test–Retest Procedures

A subset of participants completed the questionnaire twice with a target interval of two weeks. Pair matching was verified by comparing contact identifiers, timestamps, and demographic consistency between administrations. Of the 55 attempted matched pairs, one non-Saudi respondent was excluded to maintain consistent eligibility, and two pairs were excluded because their identifiers could not be verified across administrations and the demographic data did not reliably confirm a match, leaving 52 verified matched pairs.

### 3.8. Statistical Analysis

Descriptive statistics were computed for all items and domain scores, including skewness, kurtosis, and item-level floor (score = 0) and ceiling (score = 3) percentages. Floor or ceiling rates exceeding 15% were flagged per convention. Normality was evaluated through skewness and kurtosis rather than null-hypothesis tests because the Kolmogorov–Smirnov test tends to reject normality in large samples even for trivial deviations.

Content validity indices were computed from the pilot data following Lynn [15] and Polit and Beck [16]. The item-level Content Validity Index (I-CVI) was defined as the proportion of pilot participants selecting one of the two highest response options for clarity and simplicity. The scale-level index (S-CVI/Ave) was computed as the mean of the item-level I-CVIs. An I-CVI ≥ 0.78 was considered acceptable, and an S-CVI/Ave ≥ 0.90 was considered excellent [15,16].

Internal consistency was evaluated using Cronbach’s alpha, corrected item–total correlations, and alpha-if-item-deleted indices. Test–retest reliability was estimated using the two-way random-effects, absolute agreement, single-measure intraclass correlation coefficient [ICC(2,1)] as defined by Shrout and Fleiss [17], using the extended framework described by McGraw and Wong [18] and the practical guidance summarised by Liljequist et al. [19] and Mondal et al. [20]. Ninety-five percent confidence intervals were computed via the F-distribution.

CFA was used to test the a priori five-factor model in which each item loaded on only one latent factor and the five factors were allowed to correlate. The model was estimated using maximum likelihood and evaluated with the chi-square statistic, comparative fit index (CFI; acceptable ≥0.90), Tucker–Lewis index (TLI; acceptable ≥0.90), root mean square error of approximation (RMSEA; acceptable ≤0.08) with a 90% confidence interval, and standardised root mean square residual (SRMR; acceptable ≤0.08) [21].

EFA was performed as a complementary structural analysis using principal axis factoring (PAF) with Promax (oblique) rotation. Factorability was examined with the Kaiser–Meyer–Olkin (KMO) measure [22] and Bartlett’s test of sphericity [23]. Factor retention was informed by eigenvalues greater than 1.0, Horn’s parallel analysis [24] with 1000 random datasets and a 95th-percentile criterion, the scree plot, and consistency with the expected five-domain PID-5-BF structure. The full pattern matrix, variance explained, and factor correlations from the oblique rotation are reported.

Descriptive statistics, reliability analyses (Cronbach’s alpha, corrected item–total correlations, alpha-if-item-deleted), exploratory factor analysis, and intraclass correlation coefficients were computed in IBM SPSS Statistics (v28; IBM Corp., Armonk, NY, USA). Confirmatory factor analysis and Horn’s parallel analysis were conducted in R (v4.3; R Foundation for Statistical Computing, Vienna, Austria) using the lavaan and psych packages.

## 4. Ethical Considerations

Ethics approval was obtained from the Institutional Review Board of the College of Medicine, Imam Mohammad ibn Saud Islamic University, Riyadh, Saudi Arabia (Project 602/2024). Electronic informed consent was obtained from all participants before they accessed the survey. Participation was voluntary and uncompensated. All procedures were conducted in accordance with the Declaration of Helsinki and relevant institutional guidelines.

## 5. Results

### 5.1. Participant Flow and Sample Characteristics

Figure 1 summarises the derivation of the main analytic sample. Of the 431 responses received, 83 were set aside for separate analysis (pilot sample, *n* = 25; retest second administration, *n* = 58), yielding an analysis pool of 348. After sequential exclusions for non-consent (*n* = 5), non-Saudi nationality (*n* = 7), age below 18 years (*n* = 5), and missing age (*n* = 3), the final analytic sample comprised 328 Saudi adults. The mean age was 33.8 years (SD = 11.6, range 18–64); 178 (54.3%) were female, 222 (67.7%) held a university degree, 181 (55.2%) were married, and 166 (50.6%) resided in the southern region. Full characteristics are presented in Table 1.

### 5.2. Content Validity

In the pilot testing (*n* = 25; 14 male, 11 female; mean age 30.7 years; all Saudi), participants rated each item for clarity and simplicity on a four-point scale. Across 625 item-level ratings, item-level Content Validity Indices (I-CVIs) fell between 0.76 and 1.00 for clarity and between 0.80 and 0.96 for simplicity. A total of 24 of 25 items met the 0.78 I-CVI threshold for clarity, and 25 of 25 met it for simplicity. Item 20 (“I often have to deal with people who are less important than me”) had the lowest clarity I-CVI at 0.76, though its simplicity I-CVI (0.80) was acceptable. Scale-level indices were S-CVI/Ave = 0.93 for clarity and 0.94 for simplicity (Table 2), both exceeding the 0.90 standard for excellent content validity [15,16]. Only minor stylistic adjustments were needed before administering the final version in the main study.

### 5.3. Descriptive Statistics, Normality, and Floor/Ceiling Effects

Mean domain scores ranged from 4.2 (antagonism) to 6.9 (negative affectivity); the total PID-5-BF mean was 28.0 (SD = 12.4). Domain-level skewness values fell between 0.00 and 0.77, with kurtosis between −0.57 and 1.03, indicating distributions suitable for conventional parametric and latent-variable analyses, with cautious interpretation. Domain-level floor and ceiling effects were limited. However, item-level floor effects were substantial for antagonism items: Item 17 (58.2%), Item 22 (56.1%), and Item 25 (50.3%) showed the highest clustering at the minimum score. In contrast, negative affectivity items showed notable ceiling effects: Item 8 (“I worry about almost everything”; 30.5% at maximum) and Item 9 (“I get emotional easily”; 24.7% at maximum). Item-level statistics are presented in Table 3.

### 5.4. Internal Consistency and Test–Retest Reliability

Cronbach’s alpha coefficients varied from 0.70 (antagonism) to 0.78 (negative affectivity) at the domain level; the total-scale alpha was 0.89 (Table 4). Deleting any single item did not materially improve the domain-level alphas. Consistent with the multidimensional design of the PID-5-BF, the total-scale alpha should be interpreted as reflecting shared variance across correlated domains rather than as evidence of unidimensionality. The moderate domain-level alphas reflect the five-item composition of each domain and are consistent with the trade-off between brevity and internal consistency in short-form instruments [25,26].

Short-term temporal stability was examined in 52 verified matched pairs (mean age 27.6 years, SD 7.9; 86.5% male; mean retest interval 16.1 days, SD 2.4, range 13–29). ICC (2,1) estimates fell between 0.74 (detachment) and 0.85 (antagonism) at the domain level, and 0.88 (95% CI 0.80–0.93) for the total score, indicating good to excellent short-term stability. The retest subsample was heavily male-skewed and younger than the full sample; therefore, these estimates should be interpreted as preliminary.

### 5.5. Confirmatory Factor Analysis

The a priori five-factor CFA model, with each item loaded on its theoretically specified domain, was estimated using maximum likelihood on N = 328. The chi-square test was significant (χ^2^(265) = 670.46, *p* < 0.001) as expected with this sample size. RMSEA was 0.068 (90% CI 0.062–0.075), indicating acceptable approximate fit. Incremental fit indices fell below the conventional 0.90 thresholds (CFI = 0.842; TLI = 0.821), and SRMR (0.122) exceeded the 0.08 cut-off. All 25 standardised factor loadings were statistically significant (*p* < 0.001) and ranged from 0.38 to 0.79; the weakest loading was for Item 13 (“I steer clear of romantic relationships”) on detachment, and the strongest for Item 3 on disinhibition. Latent factor correlations were moderate to high (0.35–0.76), with the strongest association between negative affectivity and psychoticism. Fit indices are presented in Table 5, standardised loadings in Table 6, and latent factor correlations in Table 7.

### 5.6. Parallel Analysis and Exploratory Factor Analysis

**Figure 2:**
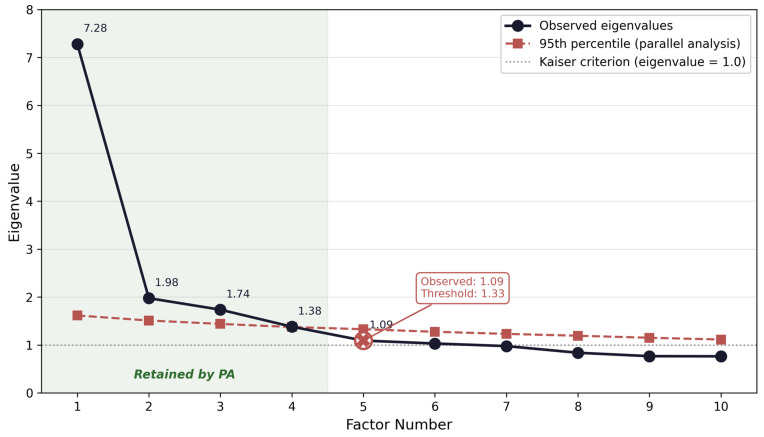
Scree plot with parallel analysis.

The correlation matrix was suitable for factor analysis (KMO = 0.889; Bartlett’s χ^2^(300) = 2773.8, *p* < 0.001). Horn’s parallel analysis with 1000 random datasets retained 4 factors; the fifth observed eigenvalue (1.09) exceeded the Kaiser criterion of 1.0 but fell below the 95th-percentile threshold from random data (1.33). This pattern is common in brief instruments with moderately correlated factors. The results are shown in Figure 2 and Table 8.

Given the convergence of the CFA model (acceptable RMSEA) with the theoretically specified five-factor structure, the EFA was specified with five factors extracted using principal axis factoring with Promax rotation. The solution explained 43.5% of the common variance. Factor correlations from the oblique rotation fell between 0.31 and 0.53 in absolute value, indicating related but distinguishable domains. Items generally loaded on their expected domains, although several cross-loadings were observed, consistent with the theoretical correlations among maladaptive personality trait domains. The full pattern matrix is presented in Table 9, and the factor correlation matrix is presented in Table 10.

## 6. Discussion

This study provides the first psychometric evaluation of the PID-5-BF in Standard Arabic that incorporates quantitative content validity data, confirmatory and exploratory factor analyses, parallel analysis, and verified test–retest reliability in a Saudi community sample. The findings support the Arabic PID-5-BF as a promising brief measure of maladaptive personality traits while identifying specific areas that require further psychometric work.

Content validity was strong, with S-CVI/Ave values of 0.93 for clarity and 0.94 for simplicity, both exceeding the 0.90 standard for excellent content validity [15,16]. Twenty-four of the twenty-five items met the 0.78 I-CVI threshold for clarity, with Item 20 falling marginally below (I-CVI = 0.76). The hierarchical social comparison expressed in this item may be conceptually or linguistically ambiguous in Arabic; future iterations could consider alternative phrasing while preserving the intended grandiosity content.

CFA of the a priori five-factor model produced mixed fit. The RMSEA was within the acceptable range (≤0.08), but CFI, TLI, and SRMR fell short of the 0.90 and 0.08 conventional thresholds [21]. Comparable patterns have been reported in other PID-5-BF validations using maximum likelihood estimation for ordinal data [27,28]. Several factors likely contribute to the below-threshold incremental fit: the small number of items per factor (five) amplifies the relative impact of measurement error; the four-point ordinal response scale attenuates fit statistics computed under continuous estimation; and method variance from shared wording is not modelled. All standardised loadings were statistically significant and ranged from 0.38 to 0.79. The weakest loading, for Item 13 on detachment, may reflect cultural differences in the salience of romantic withdrawal as an indicator of interpersonal detachment in a context where unmarried young adults commonly abstain from romantic relationships for reasons unrelated to personality pathology.

Parallel analysis, the recommended objective criterion for factor retention [24], supported four rather than five factors. The fifth factor’s eigenvalue exceeded the Kaiser criterion but fell below the 95th-percentile threshold from random data. This is not unprecedented in brief personality instruments with moderately correlated factors [27,28]. The CFA confirmed acceptable approximate fit (by RMSEA) for the five-factor model, and the five-factor EFA solution was interpretable and aligning with the theoretical structure. Although items generally loaded on their expected domains, cross-loadings were observed for several items, reflecting the theoretical overlap among maladaptive trait domains in the AMPD framework [6]. The convergence of the CFA and EFA with theoretical expectations supports retaining the five-factor interpretation while acknowledging the empirical ambiguity of the fifth factor and the below-threshold incremental fit.

Internal consistency was acceptable for a brief five-item-per-domain instrument. Domain alphas ranged from 0.70 to 0.78, and the total-scale alpha was 0.89. These values are comparable to those reported in Italian [27], Danish [28], American [29], Iranian [30], Brazilian [9], Norwegian [31], and Spanish adolescent [32] validations of the PID-5-BF. Cortina’s classic analysis [25] reminds us that alpha is influenced by the number of items and the inter-item correlations; for a five-item domain, alphas in the 0.70–0.80 range represent an acceptable trade-off between brevity and internal consistency.

Test–retest reliability was good to excellent, with ICC(2,1) values ranging from 0.74 to 0.85 at the domain level and 0.88 for the total score over a mean retest interval of 16.1 days. ICCs were computed on verified matched pairs with a correctly specified two-way random-effects, absolute agreement model [17,18,19]. However, the retest subsample was heavily male-skewed (86.5%) and younger (mean age 27.6 years) than the full analytic sample, limiting the generalisability of these stability estimates.

The pattern of item endorsement warrants particular attention. Domain-level floor and ceiling effects were limited, but item-level floor effects were substantial for antagonism items (Item 17: 58.2%; Item 22: 56.1%; Item 25: 50.3%). This pattern is supportive of the hypothesis that social desirability effects suppress endorsement of overtly antagonistic content in a collectivist cultural context where behaviours characterised as self-serving or exploitative are strongly stigmatised. In contrast, negative affectivity items showed ceiling effects (Item 8: 30.5%; Item 9: 24.7%), indicating that worry and emotional reactivity were frequently endorsed. Because no dedicated social desirability measure was administered, this interpretation remains indirect; future studies should incorporate such measures to examine response patterns directly in cross-cultural applications of the PID-5-BF.

### Strengths and Limitations

The evaluation has several strengths: it provides the first psychometric evaluation of a standard-Arabic PID-5-BF in a Saudi adult sample; it uses transparent raw-data analysis with documented inclusion criteria and recoding; and it reports a broad range of psychometric indices (content validity, internal consistency, test–retest reliability, CFA, EFA, parallel analysis, and floor/ceiling effects) with full pattern matrices and factor correlations. Several limitations must be acknowledged. First, the sample was recruited by convenience through online messaging platforms, producing an over-representation of university-educated participants (67.7%). Second, no comparator instruments, clinical diagnoses, or functional outcome measures were administered, so convergent, discriminant, and criterion validity could not be assessed; this is a fundamental limitation of the available dataset. Third, the CFA incremental fit indices fell below conventional thresholds, and parallel analysis favoured four factors; the five-factor structure, while theoretically supported, requires replication in independent samples, preferably using ordinal estimation methods (e.g., WLSMV). Fourth, the retest subsample was small (*n* = 52), demographically unrepresentative, and recruited from a convenience source; therefore, temporal stability estimates should be considered preliminary. Fifth, substantial floor effects on antagonism items suggest that self-report assessment of socially undesirable traits may be attenuated in this cultural context. Finally, only a non-clinical community sample was examined, and the psychometric properties may differ in clinical populations.

## 7. Conclusions

The standard-Arabic PID-5-BF demonstrated excellent content validity, acceptable internal consistency for a brief instrument, good short-term test–retest reliability, and a factor structure that replicated the original domain structure in this Saudi community sample. However, the structural evidence was mixed: the CFA incremental fit indices and the SRMR fell below conventional thresholds, and parallel analysis supported four rather than five factors. The present findings therefore support the Arabic PID-5-BF as a promising brief measure for research and preliminary screening, but not yet as a definitively validated instrument for routine clinical use. Future research should prioritise CFA using ordinal estimation (e.g., WLSMV) in independent samples, measurement invariance testing across gender and age [33], convergent and discriminant validity against established personality inventories, criterion validity against clinical diagnoses and functional impairment, evaluation in clinical populations, and the inclusion of social desirability measures to examine response bias in collectivist cultural contexts.

## Figures and Tables

**Table 1:** Sociodemographic characteristics of the analytic sample (N = 328).

Characteristic	Category	Value
Age (years), mean (SD)		33.8 (11.6)
Age range		18–64
Age group	≤30	154 (47.0%)
	31–40	77 (23.5%)
	>40	97 (29.6%)
Gender	Male	150 (45.7%)
	Female	178 (54.3%)
Education	School	67 (20.4%)
	University	222 (67.7%)
	Postgraduate	39 (11.9%)
Marital status	Single	136 (41.5%)
	Married	181 (55.2%)
	Divorced/widowed	11 (3.4%)
Employment	Student	38 (11.6%)
	Government	128 (39.0%)
	Private/other	27 (8.2%)
	Retired	18 (5.5%)
	Unemployed	111 (33.8%)
Region	Central	126 (38.4%)
	Southern	166 (50.6%)
	Other	36 (11.0%)

**Table 2:** Pilot content validity indices (*n* = 25).

Index	Value
Pilot participants	25
S-CVI/Ave clarity	0.931
S-CVI/Ave simplicity	0.939
I-CVI range (clarity)	0.76–1.00
I-CVI range (simplicity)	0.80–0.96
Items meeting 0.78 threshold (clarity)	24/25
Items meeting 0.78 threshold (simplicity)	25/25
Lowest-rated item (both)	Item 20

**Table 3:** Item-level descriptive statistics and floor/ceiling effects (N = 328).

Item	Domain	Text	M	SD	Skew	Kurt	Floor %	Ceil %
1	Disin	People would describe me as reckless	0.87	0.99	0.86	−0.40	46.6	9.5
2	Disin	I feel like I act totally on impulse	1.03	0.98	0.57	−0.72	36.3	9.8
3	Disin	Even though I know better, I can’t stop making ras…	0.86	0.93	0.90	−0.07	43.0	8.2
4	Detac	I often feel like nothing I do really matters	1.28	0.98	0.25	−0.94	24.7	12.8
5	Disin	Others see me as irresponsible	0.60	0.79	1.29	1.22	55.2	3.7
6	Disin	I’m not good at planning ahead	0.98	0.89	0.54	−0.55	34.5	5.8
7	Psych	My thoughts often don’t make sense to others	1.51	0.90	−0.21	−0.75	16.2	11.9
8	Negat	I worry about almost everything	1.81	1.02	−0.37	−0.99	13.1	30.5
9	Negat	I get emotional easily	1.67	1.02	−0.22	−1.06	15.9	24.7
10	Negat	I fear being alone in life more than anything else	1.17	1.05	0.44	−1.02	32.9	14.9
11	Negat	I get stuck on one way of doing things	1.14	0.93	0.46	−0.63	27.1	10.1
12	Psych	I have seen things that weren’t really there	0.92	0.96	0.73	−0.51	41.8	8.5
13	Detac	I steer clear of romantic relationships	1.46	1.08	0.07	−1.26	23.2	22.3
14	Detac	I’m not interested in making friends	0.98	0.91	0.66	−0.35	34.5	7.6
15	Negat	I get irritated easily by all sorts of things	1.15	0.92	0.46	−0.57	25.9	9.8
16	Detac	I don’t like to get too close to people	1.21	0.95	0.30	−0.88	26.8	10.4
17	Antag	It’s no big deal if I hurt other people’s feelings	0.53	0.72	1.31	1.39	58.2	2.1
18	Detac	I rarely get enthusiastic about anything	1.09	0.91	0.45	−0.65	29.6	7.9
19	Antag	I crave attention	1.13	0.91	0.28	−0.88	29.3	6.7
20	Antag	I often have to deal with people who are less impo…	1.35	0.99	0.12	−1.02	23.2	14.0
21	Psych	I often have thoughts that others say are strange	1.58	0.96	−0.20	−0.90	16.5	17.4
22	Antag	I use people to get what I want	0.54	0.70	1.29	1.62	56.1	2.1
23	Psych	I often ‘zone out’ and realize a lot of time has p…	1.36	1.03	0.18	−1.10	24.4	16.8
24	Psych	Things around me often feel unreal	1.09	0.94	0.48	−0.67	30.5	9.1
25	Antag	It is easy for me to take advantage of others	0.67	0.81	1.17	0.92	50.3	4.3

**Table 4:** Internal consistency and test–retest reliability (analytic N = 328; retest *n* = 52).

Domain	Cronbach’s α	ICC(2,1)	95% CI
Disinhibition	0.756	0.739	0.614–0.854
Negative Affectivity	0.775	0.837	0.731–0.902
Detachment	0.716	0.738	0.579–0.838
Antagonism	0.696	0.850	0.750–0.910
Psychoticism	0.730	0.825	0.710–0.894
Total	0.894	0.881	0.803–0.930

**Table 5:** CFA fit indices for the a priori five-factor model.

Index	Value	Threshold
χ^2^	670.46	
df	265	
*p* value	<0.001	
CFI	0.842	≥0.90
TLI	0.821	≥0.90
RMSEA	0.068	≤0.08
RMSEA 90% CI	0.062–0.075	
SRMR	0.122	≤0.08

**Table 6:** Standardised CFA factor loadings (N = 328).

Item	Domain	Item Content	Std. Loading
1	Disinhibition	People would describe me as reckless	0.605
2	Disinhibition	I feel like I act totally on impulse	0.707
3	Disinhibition	Even though I know better, I can’t stop making rash dec…	0.791
4	Detachment	I often feel like nothing I do really matters	0.605
5	Disinhibition	Others see me as irresponsible	0.579
6	Disinhibition	I’m not good at planning ahead	0.442
7	Psychoticism	My thoughts often don’t make sense to others	0.595
8	Negative Affectivity	I worry about almost everything	0.598
9	Negative Affectivity	I get emotional easily	0.621
10	Negative Affectivity	I fear being alone in life more than anything else	0.590
11	Negative Affectivity	I get stuck on one way of doing things	0.757
12	Psychoticism	I have seen things that weren’t really there	0.544
13	Detachment	I steer clear of romantic relationships	0.375
14	Detachment	I’m not interested in making friends	0.679
15	Negative Affectivity	I get irritated easily by all sorts of things	0.611
16	Detachment	I don’t like to get too close to people	0.637
17	Antagonism	It’s no big deal if I hurt other people’s feelings	0.585
18	Detachment	I rarely get enthusiastic about anything	0.679
19	Antagonism	I crave attention	0.482
20	Antagonism	I often have to deal with people who are less important…	0.395
21	Psychoticism	I often have thoughts that others say are strange	0.554
22	Antagonism	I use people to get what I want	0.721
23	Psychoticism	I often ‘zone out’ and realize a lot of time has passed	0.653
24	Psychoticism	Things around me often feel unreal	0.641
25	Antagonism	It is easy for me to take advantage of others	0.779

**Table 7:** Latent factor correlations from the CFA.

Factor	DIS	NA	DET	ANT	PSY
Disinhibition	1.00	—	—	—	—
Negative Affectivity	0.645	1.00	—	—	—
Detachment	0.538	0.578	1.00	—	—
Antagonism	0.629	0.352	0.522	1.00	—
Psychoticism	0.663	0.762	0.619	0.617	1.00

**Table 8:** Parallel analysis eigenvalues.

Factor	Observed Eigenvalue	95th Percentile (Random)	Retain?
1	7.280	1.616	Yes
2	1.977	1.511	Yes
3	1.737	1.440	Yes
4	1.382	1.375	Yes
5	1.092	1.329	No
6	1.031	1.276	No
7	0.977	1.232	No
8	0.838	1.193	No

**Table 9:** Promax-rotated EFA pattern matrix (principal axis factoring; N = 328).

Item	A Priori	DIS	NA	DET	ANT	PSY
1	Disin	0.508	0.028	0.082	0.005	0.132
2	Disin	0.551	−0.083	0.013	0.049	0.021
3	Disin	0.543	−0.079	0.047	−0.096	−0.006
4	Detac	0.114	−0.173	−0.236	0.005	0.120
5	Disin	0.194	−0.046	−0.144	−0.250	−0.026
6	Disin	0.263	−0.115	−0.126	0.008	−0.076
7	Psych	0.008	−0.262	−0.046	0.074	0.309
8	Negat	−0.040	−0.538	−0.098	0.139	−0.095
9	Negat	0.143	−0.457	−0.033	0.219	0.008
10	Negat	0.023	−0.497	0.140	−0.072	−0.061
11	Negat	0.119	−0.512	0.069	0.005	0.028
12	Psych	0.221	−0.208	0.018	−0.120	0.004
13	Detac	−0.046	−0.098	−0.263	0.032	0.048
14	Detac	−0.022	0.051	−0.692	0.054	−0.007
15	Negat	0.113	−0.378	−0.112	−0.055	−0.125
16	Detac	−0.140	−0.099	−0.521	−0.088	−0.048
17	Antag	0.220	0.110	−0.175	−0.346	−0.083
18	Detac	0.065	0.056	−0.485	−0.049	0.060
19	Antag	0.053	−0.220	0.130	−0.253	0.072
20	Antag	0.142	0.069	−0.066	−0.053	0.272
21	Psych	0.070	0.076	0.019	0.049	0.697
22	Antag	−0.011	0.039	−0.006	−0.621	−0.068
23	Psych	−0.156	−0.264	−0.049	−0.204	0.201
24	Psych	−0.114	−0.267	0.006	−0.178	0.216
25	Antag	0.024	0.089	0.029	−0.612	0.038

**Table 10:** EFA factor correlation matrix (Promax rotation).

Factor	DIS	NA	DET	ANT	PSY
Disinhibition	1.00	—	—	—	—
Negative Affectivity	−0.492	1.00	—	—	—
Detachment	−0.412	0.507	1.00	—	—
Antagonism	−0.501	0.411	0.462	1.00	—
Psychoticism	0.403	−0.530	−0.306	−0.500	1.00

## Data Availability

The datasets analysed during the current study are available from the corresponding author upon reasonable request. The data are not publicly available due to privacy and ethical restrictions.
